# Ageing-related bone and immunity changes: insights into the complex interplay between the skeleton and the immune system

**DOI:** 10.1038/s41413-024-00346-4

**Published:** 2024-08-05

**Authors:** Bobin Mi, Yuan Xiong, Samuel Knoedler, Michael Alfertshofer, Adriana C. Panayi, Haixing Wang, Sien Lin, Gang Li, Guohui Liu

**Affiliations:** 1 Department of Orthopedics, Union Hospital, Tongji Medical College, Huazhong University of Science and Technology, 1277 Jiefang Avenue, Wuhan, 430022 China; 2 Hubei Province Key Laboratory of Oral and Maxillofacial Development and Regeneration, Wuhan, 430022 China; 3 Division of Plastic Surgery, Department of Surgery, Brigham and Women’s Hospital, Harvard Medical School, Boston, MA USA; 4 Institute of Regenerative Biology and Medicine, Helmholtz Zentrum München, Munich, Germany; 5 Division of Hand, Plastic and Aesthetic Surgery, Ludwig - Maximilian University Munich, Munich, Germany; 6 Department of Hand-, Plastic and Reconstructive Surgery, Microsurgery, Burn Trauma Center, BG Trauma Center Ludwigshafen, University of Heidelberg, Ludwigshafen, Germany; 7 Department of Orthopaedics & Traumatology, Stem Cells and Regenerative Medicine Laboratory, Li Ka Shing Institute of Health Sciences, The Chinese University of Hong Kong, Prince of Wales Hospital, Shatin, Hong Kong, SAR 999077 P. R. China

**Keywords:** Bone, Pathogenesis

## Abstract

Ageing as a natural irreversible process inherently results in the functional deterioration of numerous organ systems and tissues, including the skeletal and immune systems. Recent studies have elucidated the intricate bidirectional interactions between these two systems. In this review, we provide a comprehensive synthesis of molecular mechanisms of cell ageing. We further discuss how age-related skeletal changes influence the immune system and the consequent impact of immune system alterations on the skeletal system. Finally, we highlight the clinical implications of these findings and propose potential strategies to promote healthy ageing and reduce pathologic deterioration of both the skeletal and immune systems.

## Introduction

Ageing is a complex and multifaceted process, resulting in functional decline and heightened vulnerability to diseases across various organ systems and tissues. Notably, the immune and skeletal systems, essential to overall health and vitality throughout one’s lifespan, exhibit pronounced vulnerability in this regard.^1^ Recent studies reveal a profound interconnection between the two systems, suggesting a finely tuned balance governing bone equilibrium and immune responses.^2^ Immunoageing, or the gradual weakening of immune functions over time, is characterized by decreased immune cell diversity and responsiveness, along with shifts in cytokine and chemokine profiles. Consequently, this culminates in a compromised immune surveillance mechanism, heightening vulnerability to a range of conditions comprising infections, cancers, and autoimmune disorders.^3,4^ Concurrently, age-related changes of the skeletal system is associated with reduced bone mass and density, increasing the risk of fractures and osteoporosis.^5^ Recent research emphasizes the mutual influence between the immune and the skeletal systems. The immune system modulates bone remodeling and regulates bone mass through the production of cytokines, chemokines, and growth factors.^6^ Inversely, bone-derived molecules such as fibroblast growth factor-23 (FGF-23) and osteopontin influence the development and function of immune cells, thereby contributing to the maintenance of immune homeostasis.^7,8^ Understanding the intricate interplay between the immune and skeletal systems is of paramount significance, as it paves the way for innovative therapeutic approaches aimed at promoting healthy ageing and countering age-related diseases.

The present review aims to provide a comprehensive overview of the current understanding of the interrelationship between age-related changes in bone and immunity. We summarize the essence of recent findings on this bone-immune nexus, elucidate the mechanisms governing bone and immune balance, and explore the potential clinical implications of these insights.

## Age-related changes in bone structure and cell ageing

A comprehensive understanding of the complex interplay between bone structure alterations and cellular ageing is essential to unravel the intricate mechanisms underlying age-related bone deterioration. This section delves into the prominent aspects of age-related changes in bone structure and the implications of cell ageing within this context.

### Age-related changes in bone structure

**Fig. 1:**
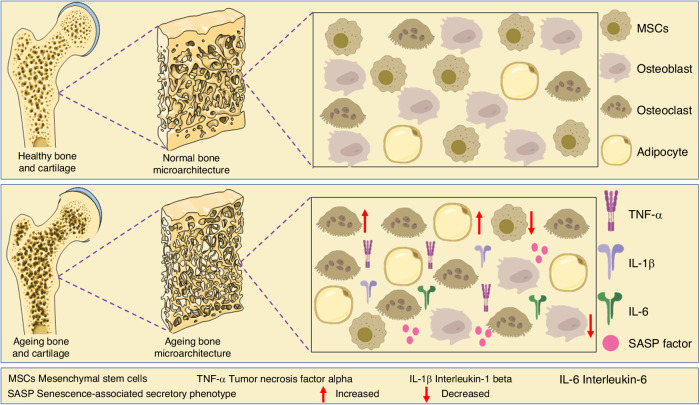
Age-related transformations in bone structure involve a decrease in bone mass and alterations in microarchitecture. The ageing process results in a reduction in the population of MSCs and osteoblasts, while there is an increase in osteoclasts and adipocytes, along with heightened levels of pro-inflammatory cytokines

Age-related changes in bone structure encompass reduction of bone mass and microarchitectural alterations, culminating in the decline of skeletal integrity with subsequent compromised mechanical properties.^9,10^ The decline in bone mineral density (BMD) is a hallmark of skeletal ageing. This reduction predominantly arises from an imbalance between bone formation and bone resorption, with the latter becoming more pronounced as age advances. This nuanced interaction is influenced by factors such as reduced osteoblast activity and an increased release of pro-inflammatory molecules, including tumor necrosis factor-alpha (TNF-α), interleukin-1 (IL-1), and IL-6. These molecular signals collectively enhance the function of osteoclasts, the cells responsible for bone resorption.^11,12^ Moreover, alterations in bone microarchitecture, including trabecular thinning and increased cortical porosity, further contribute to reduced bone strength with increasing age. These structural disruptions can be attributed to imbalances in bone remodeling, where bone resorption outpaces bone formation, resulting in a negative bone turnover. Ageing induces a myriad of alterations in the structure, composition, and function of bones, resulting in a variety of age-related diseases of the skeletal system, such as osteoporosis, osteoarthritis, and fractures.^13^ As bone ages, there are notable shifts in its composition, including alterations in the collagen matrix and mineral content. An increase in collagen cross-linking with age results in enhanced bone rigidity and diminished mechanical resilience, factors that amplify bone fragility and increase fracture risk.^14^ The mechanical properties of bones, such as strength and flexibility, are vital for maintaining overall skeletal health and mobility.^15^ Ageing leads to a decline in these properties due to a confluence of changes in bone structure, composition, and overall function (Fig. 1). Therefore, understanding the complex signaling pathways and molecular regulators governing these alterations is crucial for devising interventions to counteract age-associated bone deterioration.

### Age-related changes in bone cell ageing

Cellular ageing is increasingly recognized as a central factor in the ageing process, with a significant impact on bone health. The senescence-associated secretory phenotype (SASP), characterized by the release of proinflammatory cytokines and growth factors, creates a microenvironment able to disrupt bone remodeling and to promote chronic inflammation.

#### Bone marrow mesenchymal stem cells (BMSCs) ageing

BMSCs are pivotal players in bone homeostasis and regeneration. However, with advancing age, BMSCs undergo ageing, leading to a series of molecular alterations that undermine their regenerative potential. Telomere shortening, a key mechanism underlying cellular ageing, occurs in BMSCs, prompting DNA damage response and subsequent growth arrest.^16^ In particular, the histone chaperone nucleosome assembly protein 1-like 2 (NAP1L2) activates NF-κB, thereby inducing MSCs ageing and hindering osteogenic differentiation. Mitophagy, which is vital for cellular maintenance, regulates the ageing of BMSCs by selectively removing damaged and dysfunctional mitochondria, thereby maintaining cellular homeostasis and forestalling harmful accumulation of mitochondrial components triggering BMSC ageing. Techniques such as increasing sirtuin 1 and 3 (Sirt1 and Sirt3) expression or inhibiting LRRc17 showcase the potential for modulating BMSC ageing through mitophagy activation.^17,18^ Concurrently, p16INK4a and p53 pathways are upregulated, further reinforcing the aged status.^19^ These molecular changes collectively contribute to diminished osteogenic differentiation, decreased self-renewal capacity, and altered secretion profiles.

The clinical consequences of MSCs ageing reverberate across various facets of bone health and regenerative capacity. A decline in osteogenic potential hinders MSCs from developing into effective osteoblasts, leading to compromised bone formation and decreased mineralization, ultimately leading to age-related bone loss and posing further challenges in fracture healing and bone repair.^20^ Furthermore, the shift in secretory profile toward proinflammatory cytokines and chemokines within the SASP creates a local microenvironment that disrupts the delicate balance between bone formation and resorption. This exacerbates chronic inflammation and disrupts normal bone remodeling processes, underpinning the pathogenesis of conditions such as osteoporosis and age-related bone fractures.^21^

Therapeutic approaches targeting MSC ageing hold immense potential. Measures focusing on molecular pathways involved in MSCs ageing, such as telomere maintenance, p16INK4a inhibition, and DNA damage response modulation, could provide avenues to rejuvenate MSC functionality.^22,23^ Clinically, 1,25-dihydroxyvitamin D (1,25(OH)2 D) has been reported to suppress BMSCs ageing, bolstering anti-osteoporosis effects.^24^ Additionally, pioneering stem cell-based therapies involving the transplantation of exogenous MSCs or their derivatives, and small molecules, such as noncoding RNAs, are gaining traction to counter the detrimental effects of MSCs ageing on bone health.^25,26^

#### Osteocyte and osteoprogenitor cell ageing

Osteocytes, the primary cells in bone tissue, govern bone remodeling by detecting mechanical signals and regulating osteoblast and osteoclast functions. Ageing-related changes in osteocytes are featured by a decrease in lacunae that contain osteocytes, loss of directional orientation of lacunae, and the number and length of dendrites that connect through canniculi.^27^ The removal of osteocytes culminates in the buildup of SASP in the bone marrow, disrupting the process of osteogenesis and accelerating skeletal ageing characterized by osteoporosis.^28^ Age induces ageing in these vital cells, driven by a multitude of factors (such as diabetes and estrogen), coupled with DNA damage due to oxidative stress and weakened DNA repair systems. This results in cell cycle arrest and SASP activation, diminishing the mechanical sensing functions of osteocytes and increasing the expression of neuropeptide Y (NPY), which curbs bone adaptation.^29–31^

osteoprogenitor cell, the cells responsible for bone formation, also undergo ageing, characterized by decreased telomerase activity, telomere shortening, oxidative accumulation and DNA damage.^32^ Research on Osx1-Cre; TdRFP mice has revealed a substantial decline in the number of TdRFP-Osx1 cells in the bone marrow, implying osteoprogenitor ageing. This is marked by DNA damage, cell cycle arrest, elevated p53 and p21CIP1 levels, all of which impact bone regeneration and create a proinflammatory microenvironment.^33^ Disrupted signaling pathways such as the Nrf2/GPX4, ROS/PADI2, mTORC1/Scn1a pathways also contribute to osteoblast ageing.^34–36^ Moreover, altered microRNA profiles (such as miR-29a, miR-19a-3p) in aged osteoblasts weaken bone formation.^37,38^ The SASP, extracellular vesicles (EVs), and cytokines (e.g., RANKL released by aged osteoblasts lead to disrupted bone structure by exacerbating inflammation as well as promoting osteoclastogenesis and angiogenesis dysfunctions.^39^

The ageing of osteocyte and osteoprogenitor cell have far-reaching clinical consequences and implications. Aged osteocytes fail to adequately transmit mechanical signals, thereby affecting bone quality and rendering it more susceptible to fractures and delayed fracture healing in the elderly population. Similarly, the decline in osteoprogenitor cell functionality contributes to reduced bone formation, leading to decreased bone mass and compromised bone strength. Novel therapeutic approaches are emerging to counteract ageing of both osteocytes and osteoprogenitor cell, which comprise targeting oxidative stress and DNA damage response pathways, aiming to mitigate the initiation of ageing.^40^ Additionally, modulating main pathways and microRNA profiles could enhance osteoprogenitor cell functionality. Innovative methods using senolytic drugs, like dasatinib and quercetin, to selectively remove aged cells hold promise in restoring bone remodeling balance.^41^

#### Osteoclast ageing

Osteoclasts, derived from hematopoietic progenitor cells, are pivotal for bone remodeling through resorption of old bone tissue. With advancing age, osteoclasts undergo ageing, characterized by molecular alterations driven by factors such as an accumulation of oxidative stress and DNA damage, ultimately resulting in delayed osteoclastogenesis while preserving bone resorption capacity.^42^ Imbalances, such as a deficiency in the molecule cathepsin K and integrins, can contribute to an excess of osteoclast due to impaired apoptosis and ageing, highlighting the pivotal role of molecules in regulating osteoclast lifespan and bone homeostasis.^43^

The clinical implications of osteoclast ageing are profound. An imbalance in the number of osteoclasts can disrupt the delicate equilibrium between bone formation and resorption, causing age-related bone density changes and compromised bone remodeling. The delayed osteoclastogenesis also leads to impaired removal of damaged bone tissue, consequently hindering its replacement with new bone tissue. As a result, the bone microarchitecture is altered, rendering bones more susceptible to fractures. Moreover, the altered SASP from aged osteoclasts fosters a proinflammatory microenvironment within the bone, contributing to the chronic low-grade inflammation inherent to ageing.

Strategies targeting osteoclast ageing present promising avenues for intervention. Modulation of oxidative stress and DNA damage response pathways could delay the initiation of ageing. Additionally, therapeutic approaches able to restore signaling pathways and eliminate cytokines are crucial for osteoclast functionality, such as C5AR1, which could improve bone resorption capacity.^44^ While merely removing aged osteoclasts does not mitigate age-related loss of bone mass,^45^ novel approaches involving senolytic agents such as zoledronic acid to selectively eliminate SASP hold potential for restoring the balance between bone formation and resorption.^46^

#### Bone marrow adipocyte (BMAd) ageing

BMAd cells, once viewed simply as passive energy storers, are now understood to actively influence bone balance and act as critical energy sources for certain cells in the bone marrow. Therefore, it is essential to recognize the impact of ageing on these cells.^47^ While research on BMAd ageing remains limited, there is growing evidence suggesting that adipocytes within the bone marrow may also undergo ageing. This ageing process is influenced by various factors, including oxidative stress, DNA damage, and chronic inflammation, ultimately leading to a notable shift in their secretory profile with elevated levels of proinflammatory cytokines. Moreover, recent studies have substantiated the notion that aged adipocytes could induce secondary ageing in osteoblasts and vascular cells, thereby compounding the creation of an inflammatory microenvironment that disrupts the delicate equilibrium between bone formation and resorption.^48^ Consequently, there is an urgent need to delve deeper into the phenomenon of BMAd ageing to better comprehend its implications for bone health and overall skeletal integrity.

#### Vascular ageing

Vascular ageing, a multifaceted process influenced by factors such as telomere shortening, oxidative stress, and chronic inflammation, brings about profound transformations in the ageing vasculature.^49^ Notably, endothelial dysfunction, a hallmark of vascular ageing, is characterized by decreased levels of nitric oxide, thereby compromising vasodilation and disrupting the regulation of blood flow within the bone microenvironment. Additionally, the accumulation of collagen and loss of elastin results in arterial stiffening, further exacerbating vascular dysfunction. Previous studies have documented a significant reduction in type H vessels, arteries, and arterioles within the femur of ageing patients with osteoporosis, indicating a substantial decline in the skeletal vascular network, which could impact various physiological aspects and further exacerbate bone ageing.^50^ The bone microenvironment is impacted to a great extent by vascular ageing since diminished nutrient and oxygen supply, primarily a result of endothelial dysfunction, hinder adequate support for bone cells, ultimately impeding their regenerative potential.^51^ Consequently, impaired blood flow compromises the delivery of osteogenic precursors and growth factors to sites involved in bone formation, thereby undermining the processes of bone remodeling and repair.

To mitigate the detrimental impact of vascular ageing on bones, novel therapeutic strategies are being developed. These strategies comprise targeting oxidative stress and inflammation pathways to attenuate the initiation of ageing in vascular cells. In aged mice, enhanced HIF and Notch signaling result in the proliferation of type H vessels, subsequently leading to increased formation of trabecular bone.^52^ Moreover, innovative approaches involving senolytic agents, EVs, designed to eliminate aged cells, offer a means to restore proper vascular function, thus enhancing nutrient and oxygen supply to bone cells and potentially fostering improved bone health.^53,54^

#### Hematopoietic stem cells (HSCs) ageing

The ageing HSCs is a complex process with significant implications for immune function and overall health. Inflammation is identified as a key mediator in accelerating HSCs aging and functional decline. During inflammation, HSCs shift from anaerobic glycolysis to oxidative respiration, leading to accumulated ROS stress, triggering DNA damage, or apoptosis.^55,56^ Inflammatory cytokines such as IFN-γ have a dual role in regulating HSCs proliferation, either stimulating or hindering depending on the context. HSCs ageing results in diminished self-renewal capacity, indicated by an increased number of aged HSCs without a corresponding increase in self-renewal ability.^57,58^ Besides, HSCs ageing results in reduced differentiation potential, leading to a decrease in the formation of naive T cells and B cells. This may further negatively impact on the immune system, increasing the risk of infection, inflammation, and other relative disorders.^59^ For instance, exposure to inflammation during early to mid-life stages in mice can result in features typical of hematopoiesis in the elderly, including hemocytopenia, bone marrow cytopenia, and adipocyte accumulation.^58^ In addition, various factors, both intrinsic and extrinsic, contribute to the decline in HSCs function with age. As HSCs basically reside in the bone marrow niches, ageing of HSCs and the haematopoietic system could be linked to ageing of bones.^60^ Strategies to intervene in the aging process may have clinical relevance, especially considering the strong association between hematological pathologies and ageing. Mitochondrial metabolism, autophagy, and DNA damage response play vital roles in maintaining HSCs function and are potential targets for intervention.^61,62^

#### Chondrocyte ageing

The ageing process of chondrocytes, the unique cell type in articular cartilage, is a multifaceted phenomenon with several contributing factors during cartilage degeneration. Depletion of SIRT6, a member of the sirtuin family implicated in aging-related diseases, in human chondrocytes leads to increased DNA damage and telomere dysfunction, resulting in premature senescence.^63^ This is evidenced by decreased proliferation, elevated senescence-associated markers, and upregulated mediators for DNA damage-induced ageing. Additionally, mechanical overloading accelerates chondrocyte ageing through the downregulation of FBXW7, a key factor linking mechanical stress to chondrocyte aging.^64^ The dysregulation of specific genes, such as MMP-1 and MMP-13, further contributes to this process. Moreover, the involvement of various signaling pathways, including IL-15/JAK3/STAT5 and Notch, has been identified in the regulation of chondrocyte ageing.^65^ Clathrin-mediated endocytosis and activation of Notch signaling have been identified as contributing factors to chondrocyte ageing, while interventions targeting these pathways show promise in alleviating osteoarthritis (OA). Additionally, several non-coding RNAs are identified during chondrocyte ageing, offering potential therapeutic targets for IOA.^66,67^ Overall, understanding the molecular mechanisms underlying chondrocyte ageing is crucial for developing interventions to mitigate osteoarthritis progression.

#### Synovial cell ageing

Synovial cell ageing plays a crucial role in the progression of OA, and recent studies have revealed key mechanisms underlying this phenomenon. Aged fibroblast-like synoviocytes (FLSs) exhibit a marked increase during OA progression, accompanied by impaired autophagy leading to the upregulation of SASP. Notably, METTL3-mediated m6A modification negatively regulates autophagy in OA-FLS, specifically decreasing the expression of autophagy-related 7. Targeted METTL3 inhibition reverses FLS ageing and alleviates OA development.^68^ Additionally, inflammation-mediated tissue priming, involving synovial fibroblasts, contributes to aggravated arthritis, suggesting potential therapeutic interventions to abrogate tissue priming. The ageing synovium undergoes transcriptomic changes, with angiogenesis and fibrosis-associated genes upregulated, and FOXO1 identified as a major regulator of synovial ageing.^69^ In addition, synovial ageing promotes OA progression through immune inflammation, with identified biomarkers such as MCL1, SIK1, JUND, NFKBIA, and JUN.^70^ The synovial samples from OA patients showed strong immunoreactivity activity potential with abundant CD68^+^ macrophage, along with other immune cell infiltrate.^71,72^ For instance, the decreased omentin-1 level in synovial tissue promotes OA progression through M1 macrophage activation.^73^ Then, Grieshaber-Bouyer et al. reported that decreased CXCR1^+^ neutrophil and increased FcγRI, HLA-DR^+^ neutrophil appeared in the synovial fluid of inflamed joint.^74^ Therefore, the comprehensive understanding of synovial cell ageing and its implications in OA provides a rich foundation for developing targeted and effective therapeutic strategies.

**Fig. 2:**
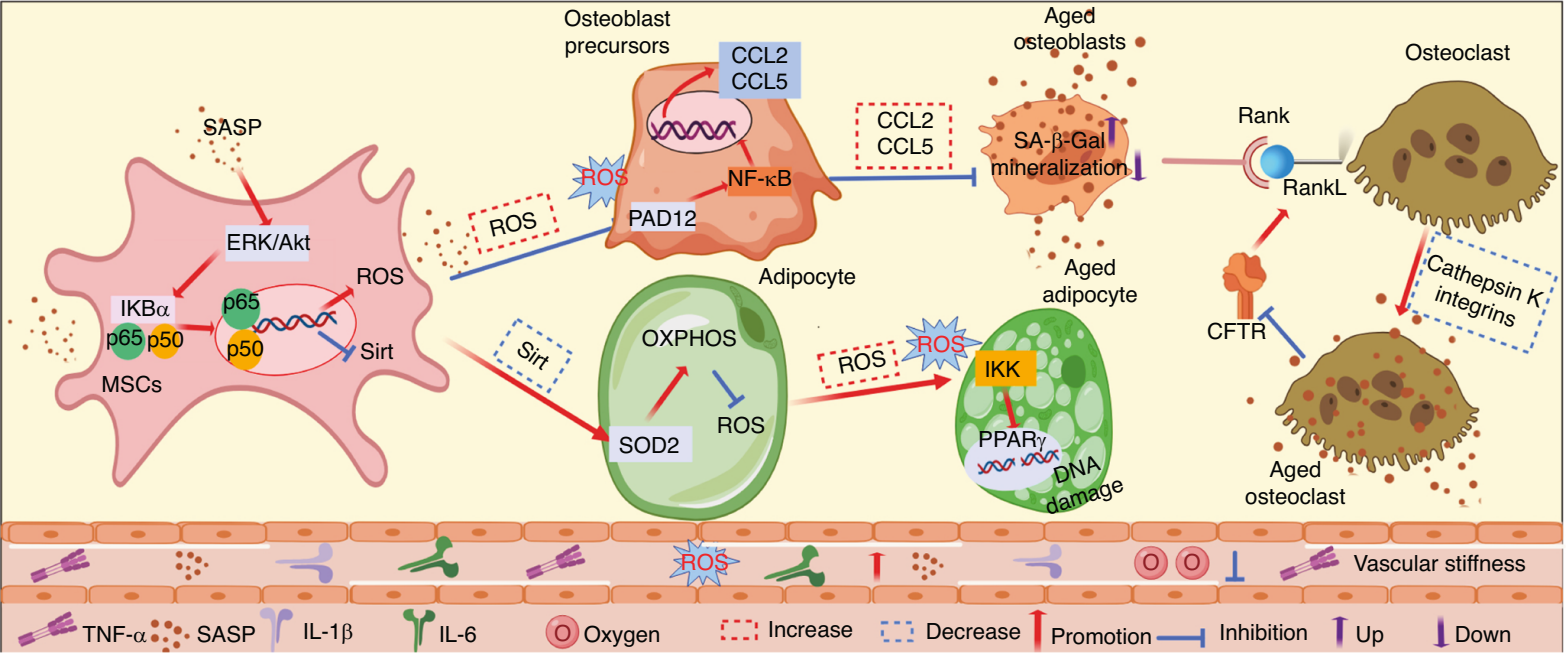
Aged-related changes on bone cell. During the ageing process, BMSCs experience ageing, which triggers the activation of NF-κB, leading to ROS production, thereby inhibits osteoblast precursor differentiation. This phenomenon is further exacerbated by elevated levels of CCL2 and CCL5, which contribute to the ageing of osteoblasts. Moreover, the decline in the levels of Sirt in aged MSCs promotes adipocyte differentiation. This results in an increase in ROS levels, leading to adipocyte ageing. This, in turn, contributes to secondary ageing in osteoblasts and vascular cells. The cumulative effect of these changes includes increased production of inflammatory cytokines and reduced oxygen availability in the vasculature, resulting in vascular stiffness and a disruption of blood flow within the bone microenvironment. Furthermore, a deficiency in molecular cathepsin K and integrins may lead to ageing in osteoclasts while preserving their bone resorption capacity

In summary, age-related changes in the bone microarchitecture, as well as alterations in bone cells (Fig. 2), lead to decreased bone strength and reduced mechanical properties. These changes synergistically contribute to decreased mobility and an increased risk of fractures and morbidity. A better understanding of the underlying mechanisms involved in the ageing process of the skeletal system and cell ageing is essential for the development of new therapeutic strategies to prevent, mitigate, and treat age-related bone diseases.

## Age-related changes in immune system

The immune system is critical for the maintenance of homeostasis and mediation of defense mechanisms against pathogens or neoplastic transformations. It is subject to continuous changes throughout life, with alterations in immune cell populations and function occurring with increasing age. Ageing of the immune system, known as immunoageing, is associated with various quantitative and qualitative changes in immune cell subsets, cytokine production, and immune responses, which all result in increased susceptibility to infections, neoplastic transformations, and autoimmune diseases. This section presents a comprehensive exploration of the intricate age-related alterations in the immune system, encompassing morphological and functional transitions of immune organs and the onset of immune cell ageing. Thoroughly understanding these changes is essential to decode the multifaceted dimensions of immunoageing and its consequent implications on bone health.

### Age-related changes in immune organs

The primary lymphoid organs, including the thymus and bone marrow, serve as central hubs for immune cell generation, maturation, and maintenance. With ageing, these critical organs undergo structural and functional changes, impacting the immune system. In childhood, the thymus is highly active, producing diverse naive T cells. However, as individuals age, the thymus shrinks and loses function, resulting in reduced production of naive T cells.^75^ This limits the diversity of the T cell receptor (TCR) spectrum, making older individuals more susceptible to infections. Thymic decline also reduces the production of regulatory T cells (Tregs), affecting immune tolerance and potentially contributing to autoimmune diseases.^76^ The bone marrow, responsible for generating various immune cells, experiences age-related alterations. Hematopoietic stem cells (HSCs) in the bone marrow change in composition and differentiation capacity.^77^ This shift towards myeloid cell production at the expense of lymphoid cells leads to an immune system reliant on innate immunity, impairing responses to pathogens requiring adaptive immunity. Changes in HSC populations can compromise overall immune competence and contribute to hematological malignancies like myelodysplastic syndromes.^78^

### Age-related changes in immune cell ageing

The initiation of immune cell ageing constitutes a central aspect of the ageing immune system. These changes are characterized by a gradual loss of immune cell functionality and the development of a proinflammatory state, both of which have far-reaching clinical ramifications.

#### T cell ageing

T cells, pivotal components of adaptive immunity, exhibit age-related ageing driven by complex molecular mechanisms.^79^

#### CD4^+^ T cell ageing

The process of CD4^+^ T cell ageing is characterized by a spectrum of intricate alterations in these immune cells unfolding with advancing age. A hallmark of this transformation includes the progressive accumulation of memory-like T cells, which exhibit alterations in their functionality and phenotype. This process coincides with the loss of expression of critical co-stimulatory molecules, which are essential for robust T cell activation and immune response. Aged CD4^+^ T cells also exhibit a shift towards a proinflammatory state, contributing to chronic inflammation seen in ageing. This altered inflammatory profile is often coupled with changes in cytokine production patterns, leading to imbalances that can further compromise immune responses. Moreover, aged CD4^+^ T cells display impaired proliferation and weakened responsiveness to antigenic stimulation, collectively diminishing the immune system’s capacity to combat infections and maintain overall health. These multifaceted changes underscore the complex nature of CD4^+^ T cell ageing and its impact on immune function. Several molecular mechanisms contribute to CD4^+^ T cell ageing, including altered gene expression, epigenetic changes, and alterations in various signaling pathways. Factors such as reduced HELIOS expression, aberrant T cell receptor (TCR) signaling, and increased expression of specific genes (e.g., DUSP4) have been implicated in driving ageing in this specific subtype of immune cells.^80,81^

Clinical consequences of CD4^+^ T cell ageing include increased vulnerability to infections, decreased responsiveness to vaccines, autoimmune diseases, and a higher risk of age-related and inflammation-associated conditions like cardiovascular disease, neurodegenerative disorders, and cancer.^82^ Addressing CD4^+^ T cell ageing is challenging, but potential therapies are emerging. Senolytic drugs aim to eliminate aged cells, potentially restoring immune function.^83^ Modulating metabolic pathways, targeting mitochondria or glycolysis, may counter metabolic changes in aged CD4^+^ T cells.^84^ Additionally, MSC-derived exosomes enriched with miRNA-21 show promise in preserving CD4^+^ T cell functionality by protecting them from oxidative damage.^85^ These strategies hold potential for mitigating CD4^+^ T cell ageing and enhancing immune vitality in ageing individuals. However, further research and clinical trials are needed to validate their effectiveness and safety across diverse ageing populations.

#### CD8^+^ T cell ageing

CD8^+^ T cell ageing, a consequence of ageing, involves reduced proliferative capacity, loss of CD28 expression, altered cytokine production, and increased ageing-associated markers like SA-β-gal. This weakens immune responses, increases infection susceptibility, reduces vaccine effectiveness, and promotes age-related diseases through chronic inflammation.^86^ The molecular mechanisms driving CD8^+^ T cell ageing involves a complex interplay of factors. Replicative ageing is caused by telomere shortening, while decreased CD28 expression reduces responsiveness to antigenic stimulation. Chronic exposure to inflammatory cytokines and oxidative stress activates stress response pathways, such as p38 MAP kinase signaling, promoting SASP.^87^ Secreting pro-inflammatory mediators amplifies inflammation and reinforces ageing, consequently impairing CD8^+^ T cell function.

Clinical consequences of CD8^+^ T cell ageing include compromised immunity against infections, reduced vaccine efficacy, and increased risk of age-related conditions like cardiovascular disease and cancer. Dysfunctional CD8^+^ T cells hinder tumor recognition and elimination, potentially promoting tumor development.^88^

Addressing CD8^+^ T cell ageing involves rejuvenating or promoting a more effective CD8^+^ T cell phenotype by targeting molecular pathways like FOXO1 or p38 MAPK signaling.^87,89^ Strategies to enhance telomere maintenance and reduce DNA damage can mitigate ageing. Immunotherapies, including immune checkpoint blockade, counter inhibitory signals contributing to CD8^+^ T cell dysfunction. Boosting the naive CD8^+^ T cell pool through regenerative medicine enhances immune responsiveness. A comprehensive approach may combine pharmacological, immunological, and regenerative strategies tailored to individuals and their associated diseases.^90^

#### Treg cell ageing

Treg cell ageing involves changes in their regulatory function within the immune system, including reduced proliferative capacity, weakened suppressive function, and altered cytokine profiles. These changes lead to chronic low-grade inflammation, impacting immune balance and homeostasis. Molecular mechanisms behind Treg cell ageing include dysregulated signaling pathways like ERK1/2 and p38, altered expression of cell-cycle-regulatory molecules (p16, p21, p53).^91^ Importantly, the downregulation of specific factors, such as DCAF1 (DDB1- and CUL4-associated factor 1), plays a critical role in Treg cell ageing by allowing increased levels of ROS and compromising Treg function.^92^ These changes result in Treg cell dysfunction, affecting immune tolerance and inflammation control.

Treg cells play a vital role in immune regulation. When these cells age, their ability to suppress immune reactions diminishes, leading to immune-related problems like autoimmune diseases, chronic inflammation, and allergies. Ageing Treg cells also lose their capacity to combat cancer cells, contributing to tumor growth.^93^ To address these issues, interventions targeting molecular pathways linked to Treg cell ageing, such as ROS regulation and key regulatory proteins like DJ-1, could be explored.^94^ Strategies to enhance mitochondrial function and metabolic activity in Treg cells may help mitigate ageing. Immunotherapies that boost Treg cell function through selective expansion or activation could restore immune balance and reduce inflammation. Innovative approaches like adoptive Treg cell therapy offer promise in replenishing Treg cells and rejuvenating the immune system. Tailored strategies for Treg cell ageing in different diseases could provide individualized solutions for immune balance and inflammation control.

#### B cell ageing

B cell ageing comprises a series of different changes. The production of new B cells decreases, leading to reduced diversity and a shift towards memory B cells. Additionally, B cells exhibit alterations in antibody production, particularly a decrease in high-affinity specific antibodies and an increase in low-affinity autoantibodies.^95^ The ageing process also affects the longevity and homing of B cells within the body, leading to changes in their distribution in different tissues.The molecular mechanisms driving B cell ageing are multifaceted. Dysregulation of critical transcription factors, such as E2A, Pax-5, and STAT5, can impair B cell development and survival.^96^ Moreover, the transcription factor Aire, responsible for promoting central T cell tolerance by mediating the presentation of self-antigens, declines in thymic B cells with age, potentially contributing to autoimmune susceptibility.^97^ Notably, the age-associated decline in spermidine levels can affect eIF5A hypusination, leading to reduced autophagy and compromised B cell function.^98^

Aged B cells exhibit reduced responsiveness to vaccinations and infections, weakening immune defense, especially against new antigens. They also contribute to autoimmune diseases through increased low-affinity autoantibodies. Age-related changes in B cell function further impact chronic inflammatory diseases.^99^ Addressing B cell ageing poses therapeutic challenges but offers hope for enhancing immune function in the elderly. Strategies to rejuvenate B cell function include targeting molecular pathways tied to ageing. For example, restoring autophagy through spermidine supplementation shows promise in rejuvenating memory B cell responses.^98^ Modulating key transcription factors like E47 can enhance B cell immune responses.^100^ Therefore, understanding the mechanisms behind B cell ageing is vital for developing targeted therapies to combat age-related immune dysfunction.

#### Macrophage ageing

Macrophage ageing involves significant changes, including reduced phagocytic activity, impaired debris clearance, compromised immunity, and SASP. These alterations have clinical implications, particularly in age-related diseases with tissue dysfunction, chronic inflammation, and weakened immunity.^101^ The molecular processes behind macrophage ageing are complex, initiated by factors like DNA damage and chronic inflammation, leading to specific signaling pathways activation, cell cycle arrest, increased mTOR activity, and altered autophagy.^102^ Aged macrophages upregulate CD47 and show changes in cytokine and chemokine expression, influenced by epigenetic modifications.^103^

Macrophage ageing has diverse clinical consequences, exacerbating age-related conditions like cardiovascular diseases, neurodegenerative disorders, and cancer, causing chronic inflammation, impaired pathogen clearance, reduced tissue repair, and pro-inflammatory molecule secretion. Addressing macrophage ageing holds promise in mitigating these consequences. Senolytic drugs can eliminate aged cells, including macrophages, thereby potentially reducing their detrimental impact. Inhibiting BRD4 and modulating macrophage phenotypes from M1 to M2 states can prevent ageing.^104^ Rejuvenating aged macrophages through autophagy enhancement or mitochondrial restoration is another option. Targeting SASP with specific cytokine or chemokine inhibitors may reduce chronic inflammation. Promoting tissue repair and regeneration can counter the negative effects of aged macrophages on age-related tissue damage through stem cell therapies or growth factors.

#### Neutrophil ageing

Neutrophil ageing brings about significant changes, including functional decline, morphological alterations, and gene expression shifts. Aged neutrophils have reduced abilities in chemotaxis, phagocytosis, and oxidative burst, making them less effective at fighting infections. Dysregulated signaling pathways and gene expression patterns underlie the molecular mechanism of neutrophil ageing, affecting chemokine and cytokine signaling, microbiome-influenced Toll-like receptor signaling, and responsiveness to inflammation.^105^

Beyond the cellular level, neutrophil ageing impacts overall health and disease susceptibility in ageing individuals. Compromised neutrophils elevate infection risk, potentially leading to more severe and prolonged infections. Reverse transendothelial migration in aged neutrophils can cause tissue damage in remote organs, increasing the risk of organ dysfunction following inflammatory events.^106^ Addressing neutrophil ageing clinically involves targeting specific molecular mechanisms. Modulating microRNAs, addressing dysregulated chemokine and cytokine signaling pathways, and microbiota interventions can potentially restore neutrophil responsiveness to inflammation.^107^ These strategies hold promise for rejuvenating neutrophil function in ageing individuals and enhancing their ability to combat infections and maintain immune balance.

#### Natural killer (NK) cell ageing

NK cell ageing involves changes in both phenotype and functionality, characterized by increased inhibitory receptors and decreased activating receptors, leading to reduced cytotoxicity, impaired cytokine secretion, and compromised immune responses. This vulnerability to infections and neoplastic transformations is accentuated by the accumulation of memory-like NK cell subsets, such as NK2.1 cells, observed in conditions like COVID-19.^108^ Molecular mechanisms behind NK cell ageing include altered gene expression profiles affecting receptors and effector functions. Activation of the SASP amplifies inflammation, impairing NK cell function. Aged cells can influence NK cell behavior and response, contributing to NK cell dysfunction.

Clinically, aged NK cells, with their reduced function, increase susceptibility to infections and neoplastic transformations, worsening disease severity. They play a role in various age-related diseases, exacerbating their progression and severity. Addressing NK cell ageing requires innovative therapeutic strategies. Immunotherapies enhancing NK cell function or replacing aged NK cells can improve immune surveillance in the elderly. Senolytic agents may rejuvenate the immune system by eliminating aged cells.^109^ Integrative therapies combining NK cell-based immunotherapies with senolytics or alternative methods offer a holistic solution to target both aged cells and dysfunctional NK cells, countering the clinical consequences of NK cell ageing and promoting healthier ageing.

#### Dendritic cells (DCs) ageing

DC ageing involves intricate molecular mechanisms influenced by various factors, including epigenetic regulation and tissue microenvironment stiffness. These changes impact DC gene expression, function, maturation, cytokine production, and immune responses.^110^ DC ageing leads to reduced immune efficacy, making individuals more vulnerable to infections, neoplastic transformations, and autoimmune diseases. It also hampers the clinical effects of immunotherapies and vaccinations in the ageing population by compromising antigen presentation, cytokine production, and T cell activation.^111^ Therapeutic strategies for DC ageing hold significant clinical potential. These approaches aim to restore or enhance DC function in ageing individuals through targeted immunotherapies like senolytics, epigenetic and metabolic modulation, and interventions maintaining tissue microenvironments conducive to optimal DC function. The goal is to mitigate the clinical consequences of DC ageing, boost immune responses, and improve health outcomes in ageing individuals.

**Fig. 3:**
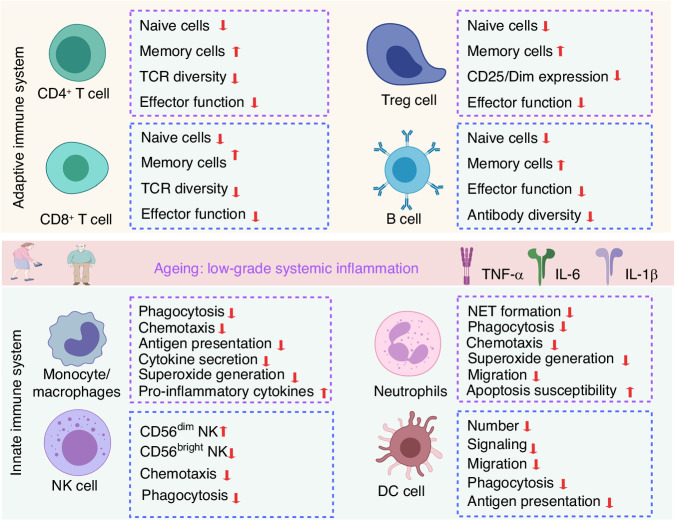
Age-related alterations affect both the adaptive and innate immune systems. In the adaptive immune system, there is a decline in the population of naive CD4^+^ T cells, CD8^+^ T cells, Treg cells, and B cells, whereas memory cells become more prevalent. In the innate immune system, the functionality of monocytes/macrophages is compromised, and neutrophils exhibit reduced capabilities in terms of NET formation, phagocytosis, and chemotaxis, along with an increased susceptibility to apoptosis. Furthermore, there is an increase in CD56^dim^ NK cells and a decrease in CD56^bright^ NK cells. The population of dendritic cells also decreases, and their anti-phagocytic abilities weaken with age

In summary, age-related alterations in the immune system encompass shifts in immune organs and the initiation of ageing of a variety of immune cells (Fig. 3). These changes have significant clinical consequences, including reduced immune responsiveness to novel pathogens and vaccinations as well as increased susceptibility to infections, heightened risk of autoimmune diseases, and chronic inflammation—all of which collectively contribute to the overall decline in health associated with increasing age. A better understanding of the underlying mechanisms involved in immunoageing and the bidirectional relationship between the immune and the skeletal system is essential for the development of new therapeutic strategies to promote healthy ageing and prevent or mitigate age-related diseases.

## Impact of ageing on the bidirectional communication between the immune system and the skeleton

The crosstalk between the immune and skeletal systems is a complex and finely tuned process, essential for the maintenance of both bone health and immune function throughout an individual’s life. However, this bidirectional communication undergoes significant changes with age. In this section, we delve into the intricate interplay between the immune and the skeletal system with increasing age, shedding light on the molecular and cellular mechanisms that underlie these interactions.

### Impact of age-related bone changes on immune systems

The bone marrow serves as the primary lymphoid organ housing hematopoietic stem cells, immune cell progenitors, and mature immune cells. However, with increasing age, the functional capacity of the bone marrow declines due to changes in the hematopoietic stem cell environment, reduced osteoblast function, and increased adipocyte deposition.^112^ These changes culminate in an impaired lymphopoiesis with reduced output of naive B and T cells.^113,114^ Age-related bone loss also reduces the available space for hematopoiesis. The aged bone marrow microenvironment shows increased levels of oxidative stress and inflammation, altering the expression of cytokines and survival factors that support immune cell maintenance. Specifically, aged HSCs exert profound effects on the immune system, contributing to immunoageing characterized by the differentiation of aged HSCs into dysfunctional immune cells, which process leads to an imbalanced myeloid/megakaryocytic differentiation bias, altering the composition of immune cells.^115^ Aged HSCs also induce inflammaging, a chronic inflammatory state marked by the release of pro-inflammatory cytokines, increasing the risk of various age-related diseases.^116^ The diminished capacity for self-renewal and regeneration in aged HSCs results in an overall decline in immune function, making individuals more susceptible to infections. Metabolic shifts in HSCs, accompanied by increased ROS, further impact their functionality. Ultimately, the consequences of aged HSCs extend to an impaired ability to mount diverse and effective immune responses, contributing to the higher incidence of infections and hematologic malignancies among the elderly. Reversing HSC aging involves multiple strategies. Transplantation of aged HSCs into a young bone marrow niche partially restores transcriptional profiles but not DNA methylation patterns, indicating intrinsic cellular aging factors that are not mitigated by a youthful environment alone.^117^ Engineering the matrix stiffness of bone marrow niche using biophysical cues like soft hydrogels can promote HSC rejuvenation by affecting MSCs behavior and improving multilineage reconstitution capacity.^118^ Additionally, manipulating soluble factors such as cytokines and implementing biomaterial-based scaffolds can enhance HSC maintenance and stemness by mimicking natural bone marrow microenvironments.^119,120^ These approaches highlight the complex interplay between extrinsic niche factors and intrinsic stem cell properties in HSC aging and rejuvenation.

**Fig. 4:**
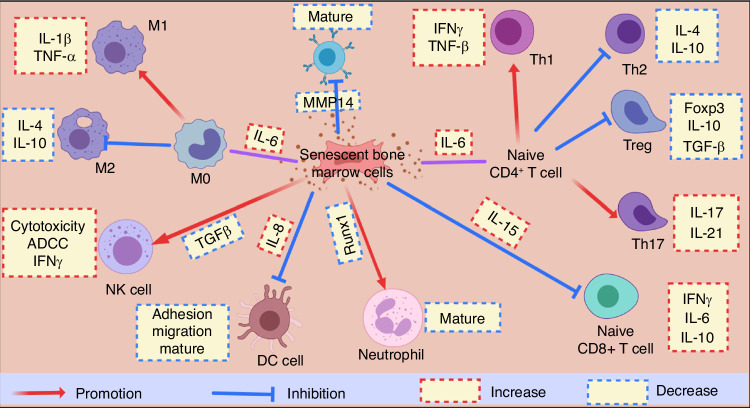
Aged bone marrow cells secrete SASP, with IL-6, IL-8, and IL-15 promoting inflammation and negatively impacting immune cell functions, including T cells, dendritic cells, B cells, NK cells, and neutrophils

In addition, aged MSCs exhibit altered behavior, particularly in the secretion of various pro-inflammatory molecules collectively referred to as the SASP, which include cytokines such as IL-6, IL-8, and IL-15, among others. The heightened levels of IL-6 play a pivotal role in influencing macrophage polarization and T cell differentiation, thereby creating a favorable environment for inflammation.^121,122^ Notably, previous research has shown that increased IL-15 levels promote the development of aged CD28^−^ CD8^+^ T cells, while decreased IL-7 levels support the maintenance of naive T cells. This imbalance contributes to the accumulation of highly differentiated effector T cells.^123^ Furthermore, the elevated levels of proinflammatory IL-6 synergize with IL-15 to drive the differentiation of CD8^+^ T cells. Additionally, IL-8, induced by aged MSCs, hinders the maturation and functioning of DCs, leading to reduced adhesion, migration, and antigen presentation by DCs.^124^ The age-related alteration in the bone marrow microenvironment in mice also leads to a reduction in B cell production and diversity of immunoglobulins. These changes are primarily attributed to extrinsic factors that affect the V(D)J recombination process in pro-B cells and hinder their progression to the pre-B cell stage, which may be related to age-associated osteoporosis and compromised marrow stem cell generation.^125^ Additionally, the loss of MMP14 on the cell surface of aged MSCs further impedes the maturation of B cells.^126^ Decreased levels of TGFβ in aged MSCs negatively affect NK cells by suppressing their natural cytotoxicity, expression of activating receptors, and IFNγ production, ultimately compromising the overall functionality of NK cells.^127^ Lastly, the deficiency of RUNX1 in aged MSCs results in heightened inflammation in neutrophils, with dysregulated Toll-like receptor 4 (TLR4) and Janus kinase/signal transducer and activator of transcription (JAK/STAT) signaling pathways originating in granulocyte-monocyte progenitors (GMPs).^128^ (Fig. 4) Downregulation of CXCL12 and proliferation-inducing ligands in the aged bone marrow impairs plasma cell survival.^129^ Overall, the aged bone marrow supports increased survival of aged and exhausted T cells while impairing central memory T cells and antibody-producing plasma cells. This skews the adaptive immune system towards a profile dominated by highly differentiated, yet dysfunctional T cells and reduces humoral immunity mediated by long-lived plasma cells. Pharmacological targeting of bone-immune interactions may help rejuvenate immunoageing by improving the maintenance of immunological memory in the bone marrow niche. For example, leverageing antioxidants could mitigate the effects of inflammageing, potentially restoring expression patterns of survival factors. Therapies that target aged T cells, modulate CXCL12, or promote plasma cell survival could also help rebalance the aged bone marrow microenvironment to better support functional immune cell populations.

In summary, understanding the age-related changes in the bone marrow microenvironment and their impact on immune cells is crucial for developing interventions that can mitigate the decline in immune function associated with aging. Furthermore, targeted strategies aimed at preserving or restoring the functional capacity of stem cells and promoting a healthy bone marrow environment may offer potential avenues for enhancing immune competence in the elderly population. As we delve deeper into the intricacies of bone marrow aging, we can explore innovative approaches to rejuvenate the immune system, potentially contributing to improved overall health and resilience in the aging population.

### Impact of age-related immune system changes on bone

**Fig. 5:**
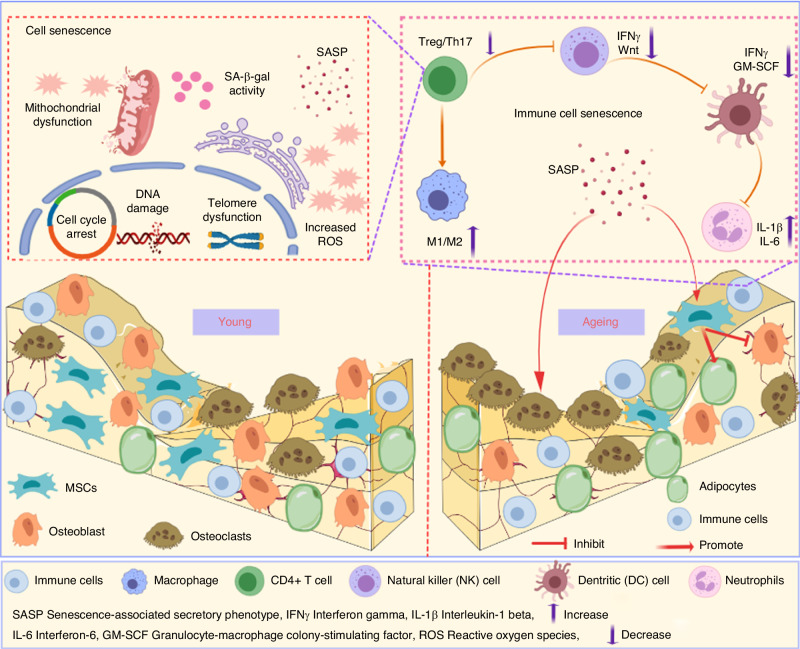
Immune cell ageing is marked by distinctive features, including mitochondrial dysfunction, DNA damage, cell cycle arrest, telomere dysfunction, and elevated levels of reactive oxygen species (ROS) and SASP factors. Moreover, aged immune cells release detrimental cytokines, contributing to an increased presence of osteoclasts and adipocytes while diminishing osteocyte numbers, consequently resulting in the development of bone fragility

The aged immune system exerts a profound and far-reaching impact on bone health. This influence manifests in the form of heightened bone resorption and diminished bone formation processes. As a consequence, this imbalance results in the gradual weakening and fragility of bones over time. The intricate interplay between immune system ageing and bone underscores the importance of understanding and addressing these age-related changes to maintain skeletal integrity and overall health (Fig. 5).

With increasing age, T cell output from the thymus declines, resulting in reduced naive T cell numbers and contracted T cell receptor diversity. Individuals with osteoporosis exhibit a higher presence of aged CD4^+^ CD28^−^ T cells compared to healthy individuals.^130^ The accumulation of aged T cells leads to increased inflammatory cytokine production, which promotes bone loss through several mechanisms. Pro-inflammatory cytokines such as TNFα and IL-6 stimulate osteoclast activity, thereby shifting the balance towards bone resorption. Further, TNFα directly inhibits osteoblast differentiation and bone formation. T cells are an important source of TNFα that drives bone loss in postmenopausal osteoporosis models.^6^ And aged cells promotes naive T cells differentiation to Th17 cell, which induce synovial fibroblasts ageing and impair OA.^131^ Inflamm-ageing impairs the anabolic response in bones to parathyroid hormone by reducing T cell production of Wnt10b.^132^ Additionally, it disrupts the bone marrow niches that support osteoprogenitors and lymphopoiesis through its effects on mesenchymal stromal cells and increased oxidative stress. Aged T cells may displace long-lived plasma cells within the bone marrow, contributing to reduced humoral immunity. Moreover, they occupy niche space required by hematopoietic stem cells, thus impairing lymphopoiesis.^59^ Furthermore, Cytomegalovirus (CMV)-specific T cells accumulate in the ageing bone marrow and, with their biased T cell receptor usage, displace polyclonal naive T cells. Ultimately, immunoageing creates an osteoimmunological environment dominated by dysfunctional effector T cells that promote bone loss. In summary, the accumulation of aged T cells and the associated inflamm-ageing process create a complex osteoimmunological environment. The impact on bone health is multifaceted, affecting both bone resorption and formation through various pathways.

The ageing process has a profound impact on neutrophils, critical players in the innate immune system, and their interactions within the bone marrow environment. Aged neutrophils exhibit impaired migration, reduced ROS production, and an increased tendency for neutrophil extracellular trap (NET) formation. NETs contain inflammatory molecules and tissue-damageing proteins. By stimulating macrophages and dendritic cells, NETs promote chronic inflammation and osteoclastogenesis, as well as directly induce osteoblast apoptosis and inhibit differentiation.^133^ The loss of CXCR2 on aged neutrophils impairs regeneration of blood vessels in the bone marrow and compromises their bone-protective functions.^134^ Additionally, impaired signals mediated by granulocyte colony-stimulating factor (G-CSF) contribute to the reduced mobilization of neutrophils within the bone marrow, further compromising their ability to respond effectively to immune challenges. Furthermore, Li et al. reported that TGFβ1^+^ CCR5^+^ neutrophils are highly increased in the bone marrow of aged mice, where they induce significant bone loss by promoting the degradation of TRAF3 in mesenchymal progenitor cells (MPCs).^135^ Aged pro-inflammatory macrophages accumulate in the bone marrow and produce TNFα, IL-1β, IL-6, and RANKL, creating an inflammatory milieu conducive to osteoclast differentiation. The impaired phagocytic capacity of aged macrophages leads to the accumulation of apoptotic cells and cellular debris within the bone marrow. This accumulation perpetuates inflammation, contributing to the overall disruption of bone homeostasis. For instance, elderly individuals face more complications during fracture healing due to inflammatory dysregulation associated with aging. Research showed that preventing macrophage infiltration at fracture sites in old mice improved healing, suggesting that age-related changes in macrophages contribute to delayed bone repair.^136,137^ Recent findings provide support for the accumulation of aged macrophages and neutrophils in the bone marrow during ageing, contributing to skeletal ageing through the activation of granulocyte-specific protein grancalcin and plexin B2 (plexinB2).^138^ In summary, the ageing-related alterations in neutrophils and macrophages collectively contribute to the dysregulation of immune responses within the bone marrow. The imbalance created by impaired neutrophil functions, altered macrophage activity, and the inflammatory microenvironment accelerates the ageing of the skeletal system, emphasizing the intricate connections between the immune and skeletal systems during the ageing process.

The ageing process exerts notable effects on B cells, influencing various aspects of their functionality and, consequently, impacting the delicate balance of bone homeostasis. Aged B cells present shortened telomeres, elevated ROS, and altered chemokine receptor profiles, all of which compromise their bone marrow homing capabilities. Further, aged B cells display decreased production of interleukin-10 (IL-10), a key anti-inflammatory cytokine, and impaired affinity maturation. This compromises the overall effectiveness of the humoral immune response. The decline in the number of long-lived plasma cells within the bone marrow further hampers humoral immunity, reducing the capacity to mount robust responses against pathogens. In addition to these immunological changes, aged B cells produce proinflammatory agents like TNFα or RANKL, as well as low-affinity autoantibodies, directly stimulating osteoclastogenesis.^139^ Impaired generation of immune complexes reduces osteoclast FcR signaling, which is crucial for maintenance of skeletal mass. Beyond effects on osteoclasts, aged B cells have intrinsic effects on osteoblasts. While B cell-derived IL-10 and the chemokine CXCL13 typically stimulate bone formation, their diminished levels during B cell ageing may suppress osteoblast function. Additionally, a decrease in the production of osteoprotegerin (OPG) by aged B cells creates an environment conducive to heightened osteoclastogenesis, further disrupting the balance between bone resorption and formation.^140^ Furthermore, the increased production of RANKL by B cells, along with their reduced numbers and impaired function, contributes to an imbalance with OPG, exacerbating bone resorption and leading to age-associated bone loss.^141^ In summary, the ageing-related changes in B cells encompass not only immunological aspects but also direct implications for bone metabolism.

DCs ageing diminishes the ability to prime naive T cells, and age-related changes in innate cells contribute to the breakdown of self-tolerance, potentially leading to autoimmunity and sterile inflammation. The presence of autoantibodies the elderly, combined with decreased levels of IL6 and TNF-α, can furthermore directly stimulate osteoclastogenesis.^142^ Age-related changes in invariant NK cells, innate lymphoid cells, and myeloid-derived suppressor cells may further disrupt the balanced regulation of immune responses and contribute to bone loss by favoring bone resorption over formation.^143^ The compromised priming ability of DCs, coupled with the breakdown of self-tolerance and alterations in key immune cell populations, creates an environment where the elderly are more susceptible to autoimmune conditions, sterile inflammation, and bone-related issues.

Therefore, the aging-associated alterations in immune cell populations, encompassing T cells, neutrophils, macrophages, B cells, and dendritic cells, collectively contribute to the intricate pathogenesis of skeletal aging. These collective changes create a complex osteoimmunological milieu, emphasizing the need for targeted therapeutic approaches to address immune dysregulation and mitigate the adverse effects of aging on skeletal health.

In conclusion, the bidirectional communication between the immune and the skeletal system is a dynamic and tightly regulated process that undergoes significant age-related changes. These changes have profound clinical implications, impacting immune responses, bone health, and the susceptibility to age-related diseases. Understanding the molecular and cellular mechanisms underlying this complex interplay is essential for developing strategies to promote healthier ageing and reduce the burden of age-related bone disorders.

## Clinical implications

### Potential interventions to promote healthy ageing of the immune system and bone

The age-related decline in immune function and bone health can have profound clinical implications, including but not limited to an increased risk of infections, neoplastic transformations, osteoporosis and OA. Therefore, identifying potential therapeutic approaches to prevent or attenuate age-related changes in the immune and skeletal system is of high scientific interest. One potential intervention is the use of immunomodulatory drugs to enhance immune function in the ageing population. For example, the use of interleukin-7 (IL-7) has been shown to enhance immune function in ageing mice and humans.^144^ Senolytic drugs, such as rapamycin, metformin, dasatinib, and quercetin, have also proven beneficial in eliminating aged cells and preventing aging (Table 1).^145–147^ Additionally, antioxidants and natural products may offer support. For instance, Geng et al. reported that pyrroloquinoline quinone inhibits oxidative stress and the release of SASP, thereby promoting osteoblastic bone formation.^40^

**Table 1:** Candidate senolytics for the treatment of aged bone and the associated clinical trials from clinicaltrials.gov/

Senolytic drugs	Cellular Effects	Immunomodulatory effects	NCT Number (https://clinicaltrials.gov/)
Rapamycin	Inhibit BMSC and Prevent ageing^18,145^	Reduce aged immune cells and improve immune function^154^	Not applicable
Metformin	Inhibits MSCs and chondrocyte ageing^146,155^	Modulate macrophage subpopulation;^156^ Suppress T cell senescence^157^	Osteoarthritis (NCT05034029; NCT05638893)
Dasatinib, & Quercetin	Eliminating the aged cells to restore MSCs function;^147,158^ Induce aged chondrogenic progenitor cells apoptosis^159^	Dampen age-specific immune responses^160^	Osteoporosis (NCT06018467)
Ruxolitinib	Suppress of SASP^151^ Delays premature aging phenotypes;^161^	Inhibit M1 macrophage polarization;^162^ Prevented hyper-inflammation^163^	Not applicable
Fisetin	Eliminating the aged osteoblasts and chondrocyte cells;^164^	Clear aged macrophages^165^	Osteoarthritis (NCT04210986; NCT04815902)
Navitoclax (ABT-263)	Promotes aged HSCs apoptosis ;^166^ Clear aged MSCs and chondrocytes^33,167,168^	Reduce IL-17^+^γδ + T cell;^169^ Ameliorate chronic inflammation of aged immune system^170^	Not applicable

Prior to initiating therapeutic agents specifically tailored to modulate the immune and skeletal system, patients should first be advised to implement lifestyle modifications, such as regular physical exercise and a healthy diet in their daily routine. Exercise has been shown to improve immune function and bone health, potentially by reducing inflammation and promoting the production of growth factors such as reticulocalbin that promote bone formation.^148^ Similarly, a healthy diet rich in calcium, vitamin D, and other nutrients can promote bone health and reduce the risk of aged-bone related disease (Table 2). Finally, the use of regenerative medicine approaches, such as stem cell therapy and tissue engineering, may hold promise for promoting healthy ageing of the immune and skeletal system.^149^

**Table 2:** The relationship between Micronutrients, immune cells and aged-related bone

Micronutrients		Impact on immune cell	Impact on bone
Vitamin	B	Serve as cofactors of enzymes to regulate energy metabolism, and other critical functions^171^	Benefit to BMD and contrast to OA progession^172,173^
	C	Acts as an antioxidant and functions as a cofactor for a number of enzymes, playing a role in enhancing the differentiation and proliferation of B- and T-cells.^174^	Promote osteogenesis and bone mineralization^175,176^
	D	Counteracts inflammation by inhibiting Th1, Th17 cell proliferation, suppression of DC cell maturation with Treg enhancement.^177^	An autocrine/ paracrine regulator of osteoblasts and bone formation^178^
	E	Assist in maintaining T cell membrane integrity, facilitating signal transduction and cell division, and regulating inflammatory mediators produced by other immune cells.^179^	Exerts anti-osteoporotic actions through alleviate IL-1, IL-6, RANKL level.^180^ However, higher vitamin E status may decrease BMD^181^
	K	Diminish T cell-mediated immunity by restraining the proliferative response and triggering apoptosis in activated T cells^182^	Enhance bone-vascular crosstalk and bone strengthen^183,184^
Amino Acids		Control immune cell differentiation and function^185^	Act as energy source and molecular pathway modulator in bone^186^
Fatty Acids	Short-Chain fatty acids	Improve memory potential of activated CD8^+^ T cells;^187^ Increases regulatory T-cell numbers and function, and decrease inflammatory cytokines expression^188^	Downregulate osteoclast genes and prevent bone loss^189,190^
	Long-Chain fatty acids	Exert effects through immune cell membrane receptors such as FFA1 and FFA4 receptors^191^	Dual modulates the viability of bone-related cells^192^

### Emerging areas of research and potential avenues for future investigation

The intricate relationship between bone ageing and immunology is a field experiencing rapid advancements, unveiling several emerging areas of research and potential avenues for deeper investigation. One promising area of research is the identification of specific immune cell populations and cytokines that regulate bone remodeling with ageing. Recent studies have identified the involvement of various immune cell populations, such as T cells, B cells, and myeloid cells in age-related bone loss.^150^ Additionally, pro-inflammatory cytokines such as IL-6, TNF-α, and RANKL have been implicated in the regulation of bone remodeling in the ageing population. Future research in this area may focus on the development of targeted interventions aiming to modulate these immune cell populations and cytokines to promote healthy ageing of the skeletal system. Another area of emerging research is the role of the cellular ageing. Aged cells accumulate with ageing and have been implicated in various age-related diseases, including osteoporosis and overall impaired function of the immune system. Recent studies have shown that aged cells can promote bone loss and impede immune functionality through various mechanisms, such as the production of pro-inflammatory cytokines. Future research in this area may focus on the development of interventions targeting these aged cells to ultimately promote healthy ageing of the immune and skeletal system.^151^ Finally, the development of novel image techniques and biomarkers may provide valuable insights into the bone-immune axis and the effects of ageing on these systems. For example, high-resolution image techniques such as micro-CT and PET can provide detailed information on bone structure and metabolism, while biomarkers such as bone turnover markers and cytokine profiles can provide information on bone remodeling and immune function.^152,153^ Future research may focus on the development of novel image techniques and biomarkers that can provide more detailed and accurate information on the bone-immune axis during ageing.

## Conclusions

In conclusion, the complex network of bidirectional communication between the skeletal and immune system, termed the bone-immune axis, plays a crucial role in the regulation of bone remodeling and immune function. The ageing process is associated with alterations in the production and activity of inflammatory cytokines, chemokines, and growth factors, leading to dysregulation of the bone-immune axis, thereby laying the foundation for age-related diseases such as osteoporosis and immune dysfunction. Further research is needed to better understand the molecular mechanisms underlying the bone-immune axis to identify novel therapeutic targets for the prevention and treatment of age-related diseases. A better understanding of the bone-immune axis will improve our ability to promote healthy ageing and improve the quality of life for the ageing population.
